# 5-*cis*-, *Trans*- and Total Lycopene Plasma Concentrations Inversely Relate to Atherosclerotic Plaque Burden in Newly Diagnosed Type 2 Diabetes Subjects

**DOI:** 10.3390/nu12061696

**Published:** 2020-06-06

**Authors:** Gemma Chiva-Blanch, Claudia Jiménez, Montserrat Pinyol, Zoe Herreras, Marta Catalán, Miriam Martínez-Huélamo, Rosa M Lamuela-Raventos, Aleix Sala-Vila, Montserrat Cofán, Rosa Gilabert, Amanda Jiménez, Emilio Ortega

**Affiliations:** 1 Department of Endocrinology and Nutrition, August Pi i Sunyer Biomedical Research Institute–IDIBAPS, Hospital Clínic of Barcelona, 08036 Barcelona, Spain; gchiva@clinic.cat (G.C.-B.); claudiajimeneztenhoevel@gmail.com (C.J.); asala@barcelonabeta.org (A.S.-V.); MCOFAN@clinic.cat (M.C.); AJIMENE1@clinic.cat (A.J.); 2 Spanish Biomedical Research Network in Physiopathology of Obesity and Nutrition (CIBEROBN), ISCIII, 28029 Madrid, Spain; lamuela@ub.edu; 3 Consorcio de Atención Primaria del Eixample (CAPSE), Grup Transversal de Recerca en Atenció Primària, IDIBAPS, 08036 Barcelona, Spain; MPINYOL@clinic.cat (M.P.); HERRERAS@clinic.cat (Z.H.); CATALAN@clinic.cat (M.C.); 4 Department of Nutrition, Food Science and Gastronomy, XaRTA, School of Pharmacy and Food Sciences, University of Barcelona, 08028 Barcelona, Spain; mmartinezh@ub.edu; 5 INSA-UB, Nutrition and Food Safety Research Institute, University of Barcelona, 08028 Barcelona, Spain; 6 Vascular Unit, Centre de Diagnòstic per l’Imatge, IDIBAPS, Hospital Clínic, 08036 Barcelona, Spain; GILABERT@clinic.cat

**Keywords:** atherosclerosis, plaque burden, lycopene, tomato, type 2 diabetes mellitus, *cis*- and *trans*-isomers

## Abstract

Diabetic subjects are at increased risk of cardiovascular disease. Atherosclerosis, the common soil of most of the cardiovascular complications, is more prevalent and extensive in this population due not only to hyperglycemia, insulin resistance, and dyslipidemia, but also to inflammation and oxidative stress. Lycopenes are bioactive compounds with antioxidant and anti-inflammatory activities mostly supplied by tomato and tomato byproducts. We investigated the association between circulating lycopenes and carotid plaque burden in diabetic patients, in a cross-sectional study in 105 newly diagnosed diabetic subjects. Atheroma plaque (wall thickness ≥ 1.5 mm), number of plaques, and plaque burden (sum of maximum heights of all plaques) were assessed by sonographic evaluation of carotid arteries. Plasma lycopenes (5-*cis*-, 9-*cis*-, 13-*cis*-, and *trans*-lycopene) were quantified by high performance liquid chromatography–mass spectrometry HPLC-MS. Atheroma plaque was observed in 75 participants, from which 38 presented one plaque and 37 two or more carotid plaques. No differences were observed in the plasmatic concentrations of lycopenes between subjects with and without atherosclerotic plaque presence. However, plaque burden was inversely associated with 5-*cis*-lycopene, all *cis*-lycopene isomers, *trans*-lycopene, and total lycopene isomers (all, *p* < 0.05). High plasma levels of lycopenes inversely relate to atherosclerotic burden. We provide novel evidence that suggests that the consumption of compounds found in tomato and tomato byproducts might be beneficial for the prevention of atherosclerosis.

## 1. Introduction

The progressive westernization of dietary patterns coupled with physical inactivity has caused a significant increase in the prevalence of obesity and a global epidemic of type 2 diabetes mellitus, expecting to affect 642 million people by 2040 [1]. The first cause of blindness, nontraumatic amputation, and terminal kidney disease is microvascular complications of diabetes, and cardiovascular disease is the first cause of mortality in this population [2]. Therefore, preventing and/or delaying the progression of cardiovascular complications in diabetes is a major public health concern.

Atheroma plaque is the hallmark of most of the cardiovascular events. In diabetes, hyperglycemia, insulin resistance and dyslipidemia lead to a proinflammatory and prooxidant status which promotes accelerated atherogenesis. In fact, atherosclerosis begins earlier, is more extensive, and progresses more rapidly than in nondiabetic individuals [3]. Together, this prompted the notion that dietary consumption of bioactive compounds with antioxidant and anti-inflammatory activities might contribute to the protection against atherosclerosis progression.

Lycopene is a red fat-soluble pigment widely found in vegetables and fruits, but more specifically in tomato and tomato byproducts. The term “lycopene” comprises different isomers suggested to differ in their origin, bioavailability and presumed healthy effects [4]. Several epidemiologic studies have evaluated the relationship between lycopene intake (without distinction among isomers), evaluated either by food frequency questionnaires [5,6,7] or serum/plasma concentrations [5,6,7,8,9,10,11,12], and ultrasound-assessed subclinical atherosclerosis with somewhat controversial results. Null [5,7,9] or inverse [6,8,10,11,12] associations have been reported between lycopene and common carotid artery intima media thickness (CCA-IMT), a very early and widely used surrogate marker of cardiovascular risk [12]. However, carotid plaque burden (defined as sum of plaque heights or plaque areas), rather than IMT, is more representative of atherosclerosis and is a better predictor of cardiovascular events [13].

To date, studies relating dietary lycopene and carotid atheroma plaque are scant [6], and none of them has been performed in a diabetic population. We therefore hypothesized that circulating lycopene would inversely relate to carotid plaque burden in diabetic patients. To address this issue, we aimed at investigating the association between lycopene consumption and subclinical carotid atheroma plaque and burden in a group of newly diagnosed diabetic patients.

## 2. Materials and Methods

### 2.1. Study Population

In this cross-sectional study, we included 105 diabetic subjects from the DIABIMCAP Study (Carotid Atherosclerosis in Newly Diagnosed Type 2 Diabetic Individuals, ClinicalTrials.gov Identifier: NCT01898572), which aims at investigating preclinical atherosclerosis in new-onset type 2 diabetes mellitus subjects [14]. The institutional ethics committee approved the protocol of the study, and it was performed in accordance with the Declaration of Helsinki. All subjects included in the study underwent a physical examination in a first visit at their primary health care center, and were selected according to inclusion and exclusion criteria. After acceptance of inclusion in the study, they signed an informed consent. Inclusion criteria were clinical and laboratory evidence of type 2 diabetes (lack of autoimmune diabetes or antiglutamic acid decarboxylase negativity in suspicious cases; and/or fasting glucose and/or HbA1c, 1999 WHO criteria, respectively), and within one year of evolution prior to inclusion in the study. Exclusion criteria were as follows: prior history of cardiovascular disease or congestive heart failure; chronic diseases such as cancer, renal failure, liver disease, or other debilitating disease; short life expectancy; history of chronic alcohol or drug abuse or dependence; and major psychiatric disease [14].

### 2.2. Clinical and Laboratory Determinations

At inclusion, age, sex, clinical, sociodemographic and anthropometric data were recorded. An Omron HEM-7223-E (Hoofddorp, The Netherlands) was used to determine blood pressure the same day of carotid ultrasound performance. Considering the fact that a high percentage of individuals were under statin treatment, known to modulate plaque regression, we calculated a statin score for statistical adjustment. The statin score was calculated as the years of cholesterol-lowering treatment multiplied by the dose of these drugs (average) normalized to simvastatin, and pretends to estimate lifelong exposure to hypolipidemic treatment.

Fasting blood samples were collected in tubes with the anti-coagulant ethylenediaminetetraacetic acid (EDTA) or serum tubes. Blood was centrifuged at 3000 rpm for 10 min at 4 °C, within 30 min of venipuncture, and plasma and serum were stored at −80 °C until analyses. Biochemical measurements were performed in the Biomedical Diagnostic Center, Hospital Clinic, Barcelona, Spain. Total cholesterol and triglycerides were measured using the COD-PAP and GPO-PAP method (cholesterol oxidase/glycerol phosphate oxidase coupled to phenol and 4-aminophenazone). High density lipoprotein (HDL) cholesterol and high sensitivity C reactive protein (hsCRP) were measured using commercially available kits by a polyethylene glycol enhanced immunoturbidimetric assay (Siemens AG, Erfurt, Germany). Low density lipoprotein (LDL) cholesterol was estimated with the Friedewald equation when patients were fulfilling the criterion of triglycerides lower than 400 mg/dL. Glucose was measured by the hexokinase method (ADVIA 2400 Chemistry Systems analyzer, Siemens AG, Erfurt, Germany).

Lipoprotein analysis in serum was performed by two-dimensional (2D) diffusion-ordered proton nuclear magnetic resonance (1H NMR) spectroscopy (DOSY), as reported [15]. In brief, 2D 1H NMR spectra were recorded on a BrukerAvance III 600 spectrometer at 310 K (Bruker BioSpin, Rheinstetten, Germany). Double stimulated echo pulse program with bipolar gradient pulses and a longitudinal eddy-current delay was performed. The relaxation delay was 2 s, the finite impulse decays were collected into 64 K complex data points and 32 scans were acquired per sample. The gradient pulse strength was increased from 5% to 95% of the maximum strength of 53.5 Gauss cm1 in 32 steps. The squared gradient pulse strength was linearly distributed. Data of lipid concentrations, very low-density lipoprotein (VLDL), LDL and HDL size and number of each type of particle, as well as subtypes of particles according to their sizes (large, medium and small) were obtained.

The extraction and isolation of lycopene isomers from human plasma was carried out avoiding light exposure, as described in Arranz et al. [16]. Briefly, plasma was extracted twice with hexane, and the organic phases were isolated, pooled, and evaporated under nitrogen flow. Finally, the residue was reconstituted with methyl tert-butyl ether (MTBE) up to 1 mL and filtered through a 13 mm, 0.45 µm polytetrafluoroethylene filter (Waters, Milford, MA, USA) into an insert-amber vial for chromatographic analysis. Samples were stored at −80 °C until analysis. The analytes of interest, namely 5-*cis*-, 9-*cis*-, 13-*cis*-, and *trans*-lycopene, were separated and identified by high performance liquid chromatography–tandem mass spectrometry (HPLC-MS/MS) according to the procedure described by Vallverdu-Queralt et al. [17], using an HP 1100 high-performance liquid chromatography system (Hewlett–Packard, Waldbronn, Germany) coupled to an API 3000 triple-quadrupole mass spectrometer (Sciex, Framingham, MA, USA) equipped with a turbo ion spray source in positive ion mode. Results are expressed as µmol/L of plasma.

### 2.3. Sonographic Assessment of Carotid Atherosclerotic Plaques

IMT and atherosclerotic plaque presence were evaluated through standardized bilateral carotid artery ultrasound imaging with an Acuson X300 ultrasound system (Siemens AG, Erfurt, Germany) equipped with a VF 10–5 linear transducer (5 to 10 MHz frequency range) [14], in the second visit. The same certified sonographer and certified reader performed all determinations, and these were quantified off-line with a semiautomatic validated software [18]. Plaques were analyzed by B-mode and color Doppler, and defined as a focal wall thickening encroaching into the arterial lumen by at least 50% of the surrounding IMT value or with thickness of at least 1.5 mm as measured from the media adventitia interference to the intima-lumen surface [19]. Plaque height was recorded either longitudinal or transversal, as appropriate. Plaque burden was calculated as the sum of maximum heights of all plaques. Variability of ultrasound carotid wall measurements was quantified by repeating 3 different days the determinations from 14 participants. Intraclass correlation coefficient was 0.92–0.96 for mean and maximum IMT (average of right and left, and maximum value from either right or left, respectively) in common, bulb, and internal carotid segments.

### 2.4. Dietary Intake

Assessment of dietary intake was performed with the 137-item semiquantitative food-frequency questionnaire validated for the PREDIMED study [20]. Subjects were asked, in an in-person interview, about dietary habits within the last year through the number and size of serving of each food item, ranging from never or almost never to >6 times a day. This questionnaire was processed with the Food Processor Nutrition and Fitness Software (ESHA Research, Salem, OR 97306, United States) to obtain dietary data. Information on tomato-based products was collected in 3 items of the questionnaire (raw tomato; “gazpacho” (a cold Spanish soup made from tomatoes, peppers, and other salad vegetables); and ketchup/fried tomato sauce).

### 2.5. Clinical and Laboratory Determinations

Normal distribution of data was assessed using graphical methods and the Shapiro–Wilk test. Variables with skewed distribution were processed by a logarithmic transformation prior to statistical analysis and are presented as antilogarithms to facilitate interpretation of the results. Descriptive data are expressed as means ± standard deviations or as absolute frequencies and percentages (categorical variables). Spearman′s correlation coefficient was used to study the association of plasmatic concentrations of the different lycopene species (and the sum of them as well) with dietary intake of tomato-based foods, cardiovascular risk factors, and with plaque burden. Associations between carotid atherosclerosis and circulating lycopenes were assessed by analysis of variance (ANOVA) and the Bonferroni post hoc test, and by constructing linear (plaque number; plaque burden) or logistic (plaque presence) regression models. The models were adjusted for age, sex, BMI, treatment with antihypertensive agents, smoking and statin score as potential confounders. Statistical significance was set at the *p* < 0.05 level in all cases. Analyses were performed using SPSS software, version 19.0 (IBM Corp., Armonk, NY, USA).

## 3. Results

### 3.1. Participants’ Characteristics

Table 1 shows the clinical characteristics of the 105 participants with new-onset type 2 diabetes mellitus included in the study. Atheroma plaque was observed in 75 (71%) participants. No differences were observed in sex, age, anthropometric or biochemical variables between subjects with and without atherosclerosis.

### 3.2. Dietary Intake

According to dietary questionnaires, no differences were observed in nutrient intake between subjects with and without atherosclerosis (Table S1). In addition, no differences were observed in tomato consumption according to the main items of the questionnaire of frequency of consumption (Table S2). Self-reported dietary consumption of tomato and tomato byproducts was directly associated to *trans*-lycopene. Furthermore, heat-treated tomato byproducts were directly associated with 5-*cis*-lycopene (*p* = 0.032) and 9-*cis*-lycopene (*p* = 0.049), and, limited to a trend with 13-*cis*-lycopene (*p* = 0.078), as can be observed in Table 2.

### 3.3. Association between Lycopene Isomers and Lipoprotein Particles

As displayed in Table S3, the number of total, large and medium VLDL particles was independently associated with carotid plaque presence and burden.

No significant associations were observed between VLDL and LDL particles and lycopene isomers. However, small HDL particles were directly associated with 13-*cis*-lycopene (Spearman’s Rho 0.230; *p* = 0.031), all *cis*-lycopenes (Spearman’s Rho 0.292; *p* = 0.015), *trans*-lycopene (Spearman’s Rho 0.240; *p* = 0.049), and total lycopene isomers (Spearman’s Rho 0.266; *p* = 0.028). A trend was also observed for 5-*cis*-lycopene (Spearman’s Rho 0.174; *p* = 0.090). These associations were maintained after multivariate adjustments (*p* = 0.034 for 13-*cis*-lycopene, *p* = 0.040 for 5-*cis*-lycopene, *p* = 0.027 for all *cis*-lycopene isomers, *p* = 0.042 for *trans*-lycopene, and *p* = 0.030 for total lycopene isomers).

### 3.4. Association between Lycopene Isomers and Atherosclerotic Burden

**Figure 1:**
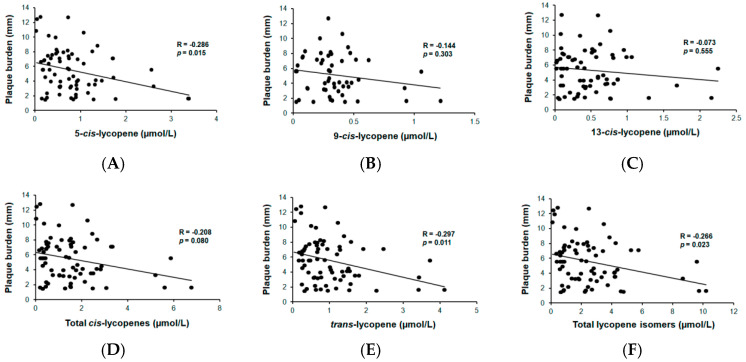
Correlations between plasmatic concentrations of 5-*cis*-lycopene (**A**), 9-*cis*-lycopene (**B**), 13-*cis*-lycopene (**C**), total *cis*-lycopenes (**D**), *trans*-lycopene (**E**), total lycopene isomers (**F**) and plaque burden. R (Rho coefficient) and *p* value from the Spearman’s correlation analyses. Plaque burden was calculated as the sum of maximum heights of all plaques. Data from *n* = 75 participants.

Among participants with atherosclerosis, one plaque was identified in 38 subjects and two or more plaques in 37 (35%) subjects. No differences were observed in the plasmatic concentrations of lycopenes between subjects with and without atherosclerotic plaque presence. However, subjects with two or more carotid plaques had significantly lower plasma concentrations of 5-*cis*-lycopene, *trans*-lycopene, and total lycopene isomers, compared to those subjects without atherosclerosis or with only one plaque (Table S4). Nonetheless, these associations disappeared after multivariate adjustment. Finally, plaque burden was inversely associated with 5-*cis*-lycopene, all *cis*-lycopene isomers, *trans*-lycopene, and total lycopene isomers, as depicted Figure 1.

These associations remained after multivariate adjustments except for all *cis*-lycopene isomers, as shown in Table 3.

## 4. Discussion

In this cross-sectional study, we examined the potential associations between plasmatic lycopene isomers and atherosclerosis in subjects with diabetes. Plasma concentrations of lycopenes were inversely related to atherosclerotic burden in these subjects, suggesting that tomato and tomato byproducts consumption, in the context of a healthy diet, may be beneficial against atherosclerosis progression.

Atherosclerosis is triggered by increased levels of cholesterol in the intima and driven by a multifactorial process including inflammation and oxidation [21]. In this setting, lycopenes have been shown to improve endothelial dysfunction by modulating the endothelial nitric oxide synthase (eNOS) pathway [3,22], and by reducing blood pressure [23]. In addition, they have been shown to induce a reduction of cytokines and adhesion molecules levels such as vascular cell adhesion molecule 1 (VCAM-1) and intercellular Adhesion Molecule 1 (ICAM-1) [12,24], and to protect against LDL oxidation [25,26] by inhibiting the myeloperoxidase activity [3] or through inhibition of 3-hydroxy-3-methylglutaryl-CoA (HMG-CoA) reductase [27]. Therefore, it seems plausible that high concentrations of circulating lycopenes, reflecting diets rich in tomato and tomato byproducts, may protect against atherosclerosis burden and progression in subjects with diabetes [28].

The associations between plasma metabolites and cardiovascular (and overall health) outcomes may be considered in the perspective of food and diet composition as a whole in spite of as the effects of a single compound or metabolite. It is known that food processing modulates the proportion of different lycopene isomers in the available foods. In this regard, both the cooking time and the presence of onions was found to increase the *cis*-isomers of lycopene in the so-called “sofrito” [29], a slow-cooked combination of tomato, olive oil, garlic and onion, widely consumed in Spain, being considered an important part of the Mediterranean Diet in this country. In fact, in healthy individuals, raw tomato consumption has been shown to decrease cellular and plasma proinflammatory biomarkers related to the onset and progression of atherosclerosis, and these effects were more pronounced after the consumption of sofrito-like cooked tomato sauce with olive oil [30], in which lycopenes are more bioavailable [17,31]. Along this line, sofrito consumption has been shown to decrease proinflammatory biomarkers such as C-reactive protein and tumor necrosis factor-alpha in healthy subjects [32] as well as in obese and overweight women [33].

Tomato consumption and, by extension, lycopene consumption, has been shown to improve the lipid profile by decreasing triglyceride and total cholesterol levels and raising HDL cholesterol in healthy individuals [30] and overweight women [34]. In type 2 diabetes subjects, tomato juice consumption decreased susceptibility of LDL to oxidation [35], and daily consumption of 200 g raw tomato for 8 weeks decreased both systolic and diastolic blood pressure and increased ApoA1 plasma concentrations [36]. We did not find any association between lycopene plasma metabolites, surrogate biomarkers of tomato consumption, and blood pressure, triglycerides, total, LDL or HDL cholesterol. Of note, we did observe a positive association between total lycopenes (and some specific isomers) and small HDL particles. Although no differences were observed in the levels of small HDL particles in subjects with different atherosclerotic burden in our study population, it is worth mentioning that these particles are inversely related to cardiovascular mortality [37].

A meta-analysis concludes that the consumption of tomato and tomato byproducts, as well as lycopene supplementation, may prevent the development of cardiovascular disease by decreasing several risk factors [38], and thus, the consumption of tomato and tomato byproducts, as well as other lycopene-rich products may significantly impact cardiovascular disease onset and progression [39]. We did not observe significant correlations between lycopene concentrations and classical cardiovascular risk factors, probably because type 2 diabetes subjects are already at increased cardiovascular risk. However, we did observe that subjects with significant atherosclerotic burden had lower plasma concentrations of 5-*cis*-lycopene, *trans*-lycopene, and total lycopene isomers, even when adjusting for cardiovascular risk factors. Since atherosclerosis is a systemic disease, detection of atheroma plaque by noninvasive techniques in the easily accessible carotid artery emerged as a surrogate marker for advanced atherosclerosis in other vascular beds, including coronary arteries. Moreover, carotid plaque is a major contributor to stroke on its own. In accordance with our results, it has been reported that elevated levels of total lycopenes are associated with decreased risk of stroke in men [40].

Our findings might contribute to shed light on the existing controversy on whether lycopene relates to atherosclerosis, since either favorable [5,7,9] or null associations [6,8,10,11,12] with CCA-IMT were previously reported. A plausible explanation for such conflict, beside differences in dietary patterns between populations, might be that no differences between lycopene isomers have been taken into account. To our knowledge, previous studies have approached this question by considering lycopene as a whole [3,12,22,23,25,26,27] or considering *trans*- lycopene species [24], but never exploring other lycopene isomers as exposures of interest. In addition, these studies were not performed in type 2 diabetes subjects, in which the effects of lycopene-rich foods consumption may differ from those observed in populations at lower intrinsic cardiovascular risk.

Our study is not exempt of limitations. First, because of its cross-sectional design, the causal link cannot be inferred. Second, the study lacks sample size calculation. Our exploratory study was performed in a subsample of individuals with diabetes embedded within a larger cross-sectional study, and, therefore, this hypothesis regarding diabetic individuals should be confirmed by further studies powered enough to uncover associations of interest or, even better, by randomized control studies. In addition, plaque calcification was not measured and would provide additional information on the relationship between lycopene consumption and severity of atherosclerosis. Finally, since the study was conducted in subjects with new diagnosis of type 2 diabetes mellitus, our results cannot be generalized to other populations. However, as a main strength of our study, we have measured objective biomarkers of intake, which plasmatic concentrations significantly correlate to self-reported consumption of tomato-based foods.

## 5. Conclusions

The plasmatic concentration of *cis*-, *trans*- and total lycopene metabolites inversely relate to plaque burden in newly diagnosed type 2 diabetes subjects at high cardiovascular risk. Long-term randomized trials are required to confirm that lycopene-rich products, such as tomatoes and their derivatives, might help in preventing or at least delaying the onset and progression of atherosclerosis, and consequently major cardiovascular events.

## Figures and Tables

**Table 1:** Clinical characteristics of the 105 participants with diabetes included in the study.

Variable	Subjects without Atherosclerosis (*n* = 30)	Atherosclerotic Subjects (*n* = 75)	*p*	Reference Interval ^1^
Females, *n* (%)	15 (50)	33 (44)	0.577	-
Age, years	58.7 ± 8.38	61.23 ± 6.83	0.112	-
BMI (kg/m^2^)	31.52 ± 5.49	30.75 ± 4.98	0.489	20–24.9
Waist (cm)	105.02 ± 11.47	104.16 ± 13.34	0.765	<100 (women) <110 (men)
Systolic blood pressure (mmHg)	129.13 ± 17.38	132.57 ± 16.46	0.343	<140
Diastolic blood pressure (mmHg)	83.17 ± 10.74	81.93 ± 9.73	0.570	<90
Glucose (mg/dL)	139.17 ± 16.41	140.18 ± 40.59	0.857	65–110
Insulin (UI/L)	20.67 ± 16.67	18.84 ± 12.15	0.538	2–15
HOMA-IR	7.26 ± 6.33	6.56 ± 4.45	0.529	<2.15
HbA1C (%)	6.92 ± 1.43	7.41 ± 1.87	0.156	3.4–5.5
hsCRP (mg/L)	0.59 ± 0.69	0.51 ± 0.53	0.488	<1.07
Triglycerides (mg/dL)	144.9 ± 94.66	151.38 ± 84.29	0.733	50–150
Total cholesterol (mg/dL)	198.83 ± 32.47	200.5 ± 44.48	0.833	148–200
HDL (mg/dL)	50.32 ± 14.84	49.09 ± 13.89	0.701	>50 (women) >40 (men)
LDL (mg/dL)	165.98 ± 50.71	174.54 ± 56.03	0.486	<130
ApoA1(mg/dL)	141.46 ± 25.02	138.01 ± 21.16	0.488	102–215
ApoB (mg/dL)	95.96 ± 23.94	104.74 ± 27.85	0.145	59–155
nonHDL (mg/dL)	146.83 ± 30.52	151.18 ± 41.87	0.559	<100
Hypertension, *n* (%)	15 (30)	41 (55)	0.665	
Dyslipidemia, *n* (%)	11 (37)	32 (43)	0.572	
Smokers, *n* (%)	4 (13)	16 (21)	0.346	
History of premature CVD, *n* (%)	2 (7)	8 (11)	0.254	
Medication, *n* (%)				
GLP-1 agonists	11 (37)	41 (55)	0.096	
Pioglitazone	11 (37)	41 (55)	0.096	
Statin score ^2^	16.02 ± 34.9	23.36 ± 46.08	0.433	

Data are expressed as mean ± SD, except for categorical variables, expressed as *n* (%). *p* from the comparison between subjects with and without carotid atherosclerosis (*t*-test for quantitative and chi square test for qualitative variables). ^1^ Reference values obtained from the core laboratory of the Hospital Clínic of Barcelona, Spain. ^2^ Statin score was calculated as the product of the duration of treatment in years by the average dose received of statin drugs standardized to simvastatin. BMI indicates body mass index; HOMA-IR, homeostatic model assessment for insulin resistance; hsCRP, high sensitivity C-reactive protein; HDL, high density lipoprotein; LDL, low density lipoprotein; CVD, cardiovascular disease; and GLP-1 denotes glucagon-like peptide-1.

**Table 2:** Correlations between self-reported dietary intake of tomato-based food items and plasmatic concentrations of different lycopene species.

Food Item	5-*cis*-Lycopene	9-*cis*-Lycopene	13-*cis*-Lycopene	*trans*-Lycopene	Sum of Lycopenes
Raw tomato, g/day					
Rho Spearman ^1^	0.189	0.190	0.154	0.184	0.209
*p* ^1^	0.073	0.072	0.145	0.082	0.046
Gazpacho, g/day					
Rho Spearman ^1^	0.179	0.166	0.139	0.185	0.194
*p* ^1^	0.089	0.115	0.187	0.080	0.065
Ketchup/fried tomato sauce, g/day					
Rho Spearman ^1^	0.227	0.210	0.188	0.215	0.206
*p* ^1^	0.032	0.049	0.078	0.041	0.053
Total tomato-based foods, g/day					
Rho Spearman ^1^	0.237	0.211	0.180	0.227	0.244
*p* ^1^	0.025	0.047	0.091	0.031	0.021

^1^ Coefficients of correlation and *p* values from the Spearman analyses.

**Table 3:** Multivariate associations of plasmatic lycopenes with carotid plaque burden in the 105 diabetic subjects included in the study.

Lycopene Measurement	B (95% CI)	*p*
5-*cis*-lycopene	−0.99 (−2.01, −0.02)	0.045
9-*cis*-lycopene	−1.26 (−4.56, 2.04)	0.445
13-*cis*-lycopene	−0.54 (−2.09, 1.01)	0.487
all *cis*-lycopene	−0.40 (−0.91, 0.11)	0.122
*trans-*lycopene	−0.86 (−1.71, −0.01)	0.047
Total lycopene isomers	−0.31 (−0.64, −0.01)	0.050

Data are presented as nonstandardized regression coefficient (B) for the association between each variable and plaque burden, with 95% confidence intervals, examined by multiple linear regression analysis adjusting for age, sex, body mass index, smoking habits (yes/no), hypertension, and statin score. Plaque burden was calculated as the sum of maximum heights of all plaques. Data from *n* = 75 participants.

## Supplementary Materials

The following are available online at https://www.mdpi.com/2072-6643/12/6/1696/s1, Table S1: Estimated nutrient intake in the 105 participants with and without atherosclerosis included in the study, Table S2: Estimated consumption of tomato and tomato byproducts in the 105 participants with and without atherosclerosis included in the study, Table S3: Lipoprotein particle number in study participants with and without atherosclerosis, and Table S4: Associations between plasmatic lycopenes with carotid plaque number in the 105 diabetic subjects included in the study.
