# Supplementary material for: 5-cis-, Trans- and Total Lycopene Plasma Concentrations Inversely Relate to Atherosclerotic Plaque Burden in Newly Diagnosed Type 2 Diabetes Subjects
Source: Nutrients. 2020 Jun 6;12(6):1696. doi: 10.3390/nu12061696 (PMC7352372; doi:10.3390/nu12061696)
Supplement: Supplementary file 1 [file nutrients-12-01696-s001.pdf]

Supplementary Materials for Chiva-Blanch G, *et al.*, *"5-cis-, trans- and Total lycopene plasma concentrations inversely relate to atherosclerotic plaque burden in newly-diagnosed type 2 diabetes subjects."*

Table S1. Estimated nutrient intake in the 105 participants with and without atherosclerosis included in the study.

Nutrient	Subjects without atherosclerosis (n=30)	Atherosclerotic subjects (n=75)	*p*
Energy (kcal/day)	2775 ± 1881	2642 ± 1989	0.770
Carbohydrates (g/day)	287.85 ± 213.76	268.29 ± 222.46	0.702
Carbohydrates (%)	38.57 ± 5.69	41.16 ± 7.28	0.216
Fiber (g/day)	33.73 ± 33.02	27.93 ± 16.34	0.399
Protein (g/day)	119.64 ± 58.64	116.83 ± 56.91	0.833
Protein (%)	19.37 ± 5.38	19 ± 5.14	0.816
Total fat (g/day)	114.22 ± 83.91	111.17 ± 102.04	0.892
Total fat (%)	37.40 ± 6.59	36.95 ± 6.76	0.824
Monounsaturated fat (g/day)	50.93 ± 37.96	47.79 ± 42.87	0.745
Monounsaturated fat (%)	16.78 ± 3.91	16.11 ± 4.12	0.588
Polyunsaturated fat (g/day)	18.95 ± 18.38	16.81 ± 16.27	0.586
Polyunsaturated fat (%)	5.78 ± 2.43	5.69 ± 2.15	0.903
Saturated fat (g/day)	33.77 ± 24.39	34.52 ± 38.86	0.927
Saturated fat (%)	11.16 ± 2.82	10.86 ± 1.84	0.706
Cholesterol (mg/day)	479.39 ± 231.36	501.39 ± 310.24	0.744
Linoleic acid (g/day)	15.53 ± 16.61	13.27 ± 16.51	0.557
Non-marine n3 fatty acids (g/day)	1.82 ± 2.01	1.66 ± 1.81	0.714
Marine n3 fatty acids (g/day)	1.02 ± 0.69	1.05 ± 0.73	0.881
Trans fatty acids (g/day)	0.83 ± 0.61	0.58 ± 0.42	0.405
Vitamins			
Retinol equivalents (µg/day)	1551 ± 1023	1684 ± 1005	0.571
D (µg/day)	6.91 ± 5.41	7.02 ± 5.28	0.925
E (mg/day)	13.12 ± 10.53	11.17 ± 8.01	0.340
C (mg/day)	297.71 ± 267.86	219.04 ± 143.91	0.166
B1 (mg/day)	2.69 ± 1.87	2.45 ± 1.54	0.521
B2 (mg/day)	2.31 ± 1.26	2.35 ± 1.12	0.885
B3 (mg/day)	50.99 ± 24.44	48.58 ± 23.51	0.662
B6 (mg/day)	3.02 ± 1.95	2.65 ± 1.28	0.293
B9 (µg/day)	501.79 ± 429.17	399.3 ± 192.57	0.251
B12 (µg/day)	10.88 ± 5.37	11.85 ± 5.88	0.468
Minerals			
Calcium (mg/day)	1253 ± 764	1174 ± 621	0.607
Iron (mg/day)	22.36 ± 15.77	19.41 ± 9.74	0.281
Sodium (mg/day)	3227 ± 2119	3028 ± 1895	0.661
Magnesium (mg/day)	512.69 ± 378	442.55 ± 219.23	0.270
Potassium (mg/day)	5576 ± 3814	5014 ± 2512	0.410
Iodine (µg/day)	271.82 ± 161.29	334.2 ± 238.09	0.223
Selenium (µg/day)	121.28 ± 71.51	114.38 ± 63.32	0.651

Zinc (mg/day)	15.23 ± 8.26	14.52 ± 7.27	0.687
Phosphorous (mg/day)	2223 ± 1143	2117 ± 1047	0.670

p values from the comparison between subjects with and without atherosclerosis (t-test for independent samples).

Table S2. Estimated consumption of tomato and tomato byproducts in the 105 participants with and without atherosclerosis included in the study.

Food Item	Subjects without atherosclerosis (n=30)	Atherosclerotic subjects (n=75)	*p*
Raw tomato (g/day)	52.66 ± 48.06	83.41 ± 95.55	0.129
Gazpacho (g/day)	51.76 ± 106.83	27.59 ± 67.33	0.292
Ketchup / fried tomato sauce (g/day)	0.39 ± 0.59	0.56 ± 0.88	0.379
Total tomato-based foods (g/day)	102.77 ± 127.12	111.13 ± 117.65	0.765

p values from the comparison between subjects with and without atherosclerosis (t-test for independent samples).

Table S3. Lipoprotein particle number in study participants with and without atherosclerosis.

Particle number (nmol/L)	Subjects without atherosclerosis (n=30)	One plaque (n=38)	Two or more plaques (n=37)	p^1	p^2
VLDL	59.93 ± 23.07^a	63.76 ± 34.12^b	86.27 ± 42.68^c	0.008	0.001
Large VLDL	1.68 ± 1.04^a	2.13 ± 1.95^b	3.48 ± 2.83^c	0.004	0.001
Medium VLDL	8.59 ± 4.56^a	9.85 ± 7.61^b	14.28 ± 10.1^c	0.017	0.003
Small VLDL	53.84 ± 28.15^a	51.78 ± 25.49^a	72.76 ± 39.12^c	0.015	0.005
LDL	1479.63 ± 381.76	1528.59 ± 385.42	1557.83 ± 436.26	0.752	0.179
Large LDL	175.93 ± 90.41	174.33 ± 83.14	184.27 ± 142.07	0.924	0.798
Medium LDL	553.35 ± 226.43	585.43 ± 230.36	603.4 ± 339.08	0.772	0.309
Small LDL	750.35 ± 198.59	768.84 ± 248.01	770.15 ± 240.74	0.935	0.320
HDL	28.69 ± 8.62	29.59 ± 5.86	27.67 ± 6.38	0.523	0.403
Large HDL	1.29 ± 0.91	1.01 ± 1.06	0.81 ± 0.71	0.134	0.089
Medium HDL	7.1 ± 3.3	7.08 ± 3.93	6.15 ± 2.94	0.457	0.526
Small HDL	20.3 ± 10.1	21.34 ± 7.08	20.7 ± 7.59	0.877	0.786

Data are shown as mean ± standard deviation. *p* values from the comparison ([1]one-way ANOVA, and [2]adjusting by age, sex, body mass index, smoking habits (yes/no), hypertension and statin score). Different superscript letters in rows indicate significant differences between values in rows, according to plaque burden (Bonferroni *posthoc* test). Statin score was calculated as the product of the duration of treatment in years by the average dose received of statin drugs standardized to simvastatin.

Table S4. Associations between plasmatic lycopenes with carotid plaque number in the 105 diabetic subjects included in the study.

**Lycopene isomers**	**Less than 2 plaques (n=68)**	**2 or more plaques (n=37)**	p^1	p^2
5-*cis*-lycopene (µmol/L)	0.53 ± 0.38	0.44 ± 0.46	0.092	0.447
9-*cis*-lycopene (µmol/L)	0.38 ± 0.24	0.3 ± 0.23	0.075	0.287
13-*cis*-lycopene (µmol/L)	1.13 ± 0.85	0.82 ± 0.73	0.026	0.177
all-*cis*-lycopene (µmol/L)	0.92 ± 0.71	0.65 ± 0.55	0.022	0.149
trans-lycopene (µmol/L)	1.66 ± 1.33	1.26 ± 1.21	0.063	0.281
Total lycopene isomers (µmol/L)	2.79 ± 2.16	2.04 ± 1.93	0.033	0.19

p values from the comparison ([1]*t*-test for unrelated samples, and [2]adjusting by age, sex, body mass index, smoking habits (yes/no), hypertension and statin score). Statin score was calculated as the product of the duration of treatment in years by the average dose received of statin drugs standardized to simvastatin.
